# Structure-guided engineering and molecular simulations to design a potent monoclonal antibody to target aP2 antigen for adaptive immune response instigation against type 2 diabetes

**DOI:** 10.3389/fimmu.2024.1357342

**Published:** 2024-03-08

**Authors:** Abbas Khan, Muhammad Ammar Zahid, Anwar Mohammad, Abdelali Agouni

**Affiliations:** ^1^ Department of Pharmaceutical Sciences, College of Pharmacy, QU Health, Qatar University, Doha, Qatar; ^2^ Department of Biochemistry and Molecular Biology, Dasman Diabetes Institute, Kuwait City, Kuwait

**Keywords:** AP2, CA33, antibody, structural engineering, docking, simulation, free energy calculation

## Abstract

**Introduction:**

Diabetes mellitus (DM) is recognized as one of the oldest chronic diseases and has become a significant public health issue, necessitating innovative therapeutic strategies to enhance patient outcomes. Traditional treatments have provided limited success, highlighting the need for novel approaches in managing this complex disease.

**Methods:**

In our study, we employed graph signature-based methodologies in conjunction with molecular simulation and free energy calculations. The objective was to engineer the CA33 monoclonal antibody for effective targeting of the aP2 antigen, aiming to elicit a potent immune response. This approach involved screening a mutational landscape comprising 57 mutants to identify modifications that yield significant enhancements in binding efficacy and stability.

**Results:**

Analysis of the mutational landscape revealed that only five substitutions resulted in noteworthy improvements. Among these, mutations T94M, A96E, A96Q, and T94W were identified through molecular docking experiments to exhibit higher docking scores compared to the wild-type. Further validation was provided by calculating the dissociation constant (K_D_), which showed a similar trend in favor of these mutations. Molecular simulation analyses highlighted T94M as the most stable complex, with reduced internal fluctuations upon binding. Principal components analysis (PCA) indicated that both the wild-type and T94M mutant displayed similar patterns of constrained and restricted motion across principal components. The free energy landscape analysis underscored a single metastable state for all complexes, indicating limited structural variability and potential for high therapeutic efficacy against aP2. Total binding free energy (TBE) calculations further supported the superior performance of the T94M mutation, with TBE values demonstrating the enhanced binding affinity of selected mutants over the wild-type.

**Discussion:**

Our findings suggest that the T94M substitution, along with other identified mutations, significantly enhances the therapeutic potential of the CA33 antibody against DM by improving its binding affinity and stability. These results not only contribute to a deeper understanding of antibody-antigen interactions in the context of DM but also provide a valuable framework for the rational design of antibodies aimed at targeting this disease more effectively.

## 1 Introduction

Diabetes mellitus (DM) is considered the oldest chronic disease that is characterized by high glucose levels in the blood. It mainly occurs due to the scarcity of insulin production and can be classified into two types: type 1 (T1DM) and type 2 (T2DM) DM (1, 2). The condition arises from the destruction of pancreatic beta-cells which consequently cannot produce insulin. In T2DM, insulin production is decreased but not completely abolished. The delay in diagnosis or management of diabetes may lead to serious complications such as diabetic neuropathy, retinopathy, diabetic foot ulcer, and cardiovascular diseases. DM is also considered as a socioeconomic burden and recent data revealed that by 2049, there will be 629 million people suffering from DM worldwide (3). The major contributing risk factors in the development of this condition include genetic predisposition, obesity, and a sedentary lifestyle. The distorted metabolic functioning and regulation of the adipose tissue are also considered another important aspect contributing to the pathophysiology of DM (4, 5).

Adipose tissue is an endocrine organ that maintains the homeostasis of various other tissues such as the brain, pancreas, and liver (6). Adipocytes respond to metabolic and immune cues by mobilizing their fat stores through lipolysis and by secreting a variety of hormones known as adipokines (7). Such signals interact with the target tissues to regulate several important processes such as glucose or insulin production. Integration of systematic metabolic regulation with adipocytes is primarily controlled by a (FAPB4) fatty acid binding protein 4 or aP2 (8). Since its discovery, the role of aP2 has been depicted in lipid metabolism and the pathogenesis of several metabolic diseases such as atherosclerosis, fatty liver, and diabetes (9–11). Improved liver function, increased sensitivity to insulin, and reduced fatty liver have been reported in mice deficient with aP2 protein thus showing the essential role of this protein in chronic metabolic disorders. The connection between aP2 and T2DM is further corroborated by genetic investigation studies conducted in diverse populations (12). These studies have shown that individuals with a rare haplo-sufficiency mutation in the *aP2* gene experience metabolic and cardiovascular advantages (13). This finding further confirms the involvement of aP2 in the pathogenesis of metabolic diseases. Being an intracellular protein, aP2 also acts as an active adipokine, a peptide that is secreted by adipose tissue that regulates hepatic glucose production and systematic glucose homeostasis. It has also been reported that aP2 contributes to insulin resistance as its serum levels are significantly elevated in obese mice and T2DM (14). In human-based investigations, the role of aP2 was observed in metabolic and cardiovascular disorders. Nonetheless, in a population-based study, reduced expression of aP2 was found to protect against cardiovascular disease and diabetes. Taken together, these findings underline that the biological and hormonal roles of aP2 are evolutionarily conserved and hold relevance in the context of human pathophysiology. Furthermore, the presence of secreted aP2 indicates a robust and promising therapeutic target for the development of therapeutics for diabetes (10, 15). Additionally, this paradigm-shifting evidence about aP2 biology underscores the potential for designing novel therapeutics based on anti-aP2 monoclonal antibodies (mAb) and offers potential solutions to the existing challenges in diabetes treatment (16).

Targeting aP2 therapeutically is a formidable task; however, Burak et al. identified a mAb, CA33, specifically targeting aP2 that was reported to improve glucose metabolism, increase insulin sensitivity, reduce fat mass, and ameliorate liver steatosis in obese mouse models (16). They reported that the novel mAb, CA33, binds to the aP2 through a direct interaction with the light chain and an indirect interaction with the heavy chain. Improving the specificity and binding of CA33 may yield better therapeutic outcomes and elicit stronger immune response. Therefore, using state-of-the-art computational methods is a promising approach to engineer therapeutic proteins for improved bindings. *In silico* saturation, mutagenesis offers a faster and more accurate way to improve the binding by inducing specific mutations. For instance, such methods have been used to engineer different proteins in different diseases such as stomach ulcers, cancer, and SARS-CoV-2 (17–20).

Computational methods have greatly accelerated the identification and development of therapeutic agents against various diseases (21, 22). As proof of the principle of this therapeutic direction, the current study uses in silico mutagenesis approaches by employing the graph signature-based algorithm to determine the impact of novel substitutions on the binding of CA33 with aP2. We resolved the mutated structures by using Chimera software and the interaction of the mutated CA33 with aP2 was predicted through the HADDOCK algorithm. A mutational landscape of 57 mutants was constructed which revealed that only 4 substitutions were able to improve the binding. The mutations designed to enhance affinity were subsequently examined through the utilization of dissociation constant calculations and molecular simulations. These analyses have confirmed the efficacy of the four most prominent mutants, namely T94M, T94W, A96Q, and A96GE, in their ability to enhance the binding affinity of CA33 with aP2. These mutant variants may be deemed suitable for experimental verification in the context of therapeutic applications.

## 2 Materials and methods

### 2.1 Structure retrieval, preparation, and interface analysis

The crystallographic coordinates of the aP2-CA33 complex were retrieved from the Protein Databank (RCSB) using the accession number 5C0N. the native structure contains three chains including the aP2 which comes in direct contact with the light (L) chain of the antibody and a heavy (H) chain of the antibody which interacts indirectly with the aP2 (23). The structures were assessed before further processing and the L chain has some missing residues so Modeler was used to model the missing loops. The structure was minimized and prepared in Chimera using the Conjugate gradients and steepest descent algorithms to relax the contacts and address deformity (24). The final prepared structure was submitted to PDBsum and analyzed for the contacts using PyMOL visualization software. The interface residues were retrieved using the PDBsum and PyMOL consensually (25, 26).

### 2.2 Graph-based signature algorithm for antibodies modeling

For the flexible and robust recognition and binding of the CA33 antibody by aP2, we employed a computational algorithm, graph-based signatures, available as mCSM-Ab2 (http://structure.bioc.cam.ac.uk/mcsm_ab) which uses experimental data to predict the impact of a particular mutation on the binding of antigen and antibody (27). The interface residues were scanned for predicting the essential contacts which revealed three residues important for recognition while the other three contacts are supplementary. We generated a mutational landscape of 57 mutants by replacing the Glu27, Thr94, and Ala96 with the remaining 19 amino acids to understand the impact on stability and binding affinity. The two contacts Tyr92 and Asp28 were kept the same as they are required for the recognition of the antigen. Among the 57 mutants only top mutations that affect the overall binding (increase) were selected for subsequent analysis. The top-scoring residues that increase the binding of the antibody were modeled in Chimera using the Dunbrack rotamers library based on the proper sidechain torsion (chi) and probability value (24). For optimization purposes, rotamer sampling and side-chain flexibility were applied.

### 2.3 Antigen-Ab docking using HADDOCK

To model the biomolecular complexes of the antigen (aP2) and antibodies, we used a high ambiguity-driven protein-protein docking (HADDOCK) algorithm. This approach utilized the biophysical and biochemical data to model the interactions and gives the results based on chemical shift perturbation data obtained from NMR titration experiments of mutagenesis data. The obtained information is then incorporated into the docking process such as Ambiguous interaction Restraints (AIRs). An AIR is specifically characterized as an uncertain distance constraint involving all residues that have been identified as participants in the interaction. For docking the protonation states were set as default (*“authohis = true”*). The Z-positioning restraints were also set to default as experimental restraints. The surface contact restraint was set as *“surfrest = true”* while the dihedral angles were also set as default. The top-scoring complexes based on the HADDOCK docking score and Z-scores were retrieved analyzed and subjected to interactions and subsequent analysis (28). The residues Glu27, Asp28, Tyr92, Thr94, and Ala96 were selected as the interface residues for the heavy and light chain of CA33 while the residues Lys9, Leu10, Val11, Lys37, and Glu129 were selected as the active residues for aP2 interaction.

### 2.4 Determination of the binding strength through dissociation constant prediction

The dissociation constant is an essential aspect of determining the pharmacological potential of antigen-antibodies complexes modeling and the results provide essential insights into the impact of a particular mutation on the recognition and binding. We used PRODIGY, a contact-based predictor, for modeling the binding strength of the native and mutated CA33 antibody with aP2 (29). The Prodigy server is the most widely and highly accurate server used for predicting the dissociation constant of a macromolecular complex. The server uses the interatomic contacts with 5.5Å and combines them with the non-interacting surface (NIS) to derive essential knowledge regarding the binding strength of K_D_.

### 2.5 All-atoms molecular simulation and analysis

We assessed the dynamic characteristics of the wild-type, T94M, T94W, A96Q, and A96E complexes in conjunction with E4R using the AMBER 21 software. To prepare the system, we employed the “tleap” module from AmberTools to generate topology and coordinate files. Missing atoms and hydrogens were added via the LEaP builder tool. To achieve charge neutrality, we introduced counterions using the AddToBox module, and for solvation, we incorporated an optimal point charge (OPC) model of the water box using the SolvateBox module. Initially, we conducted an energy minimization of the system, employing both the steepest descent and conjugate gradient algorithms. This minimization process ran for 10,000 and 8,000 steps or until the energy change became less than 0.1 kcal/mol. Subsequently, we subjected the system to a 10 ns equilibration period. During the initial 100 ps of equilibration, we applied Langevin dynamics with a collision frequency of 1.0 ps^-1^ to raise the system’s temperature from 0K to 300 K. Following this, we maintained a constant pressure of 1 atm using the Parrinello-Rahman barostat for 1 ns. This was succeeded by sustaining a constant temperature of 300K through Langevin dynamics for an additional 1 ns. Finally, a 7 ns equilibration simulation was performed utilizing an NPT ensemble with PME electrostatics and a non-bonded cutoff of 10 Å. After achieving equilibration, we conducted a 300 ns production simulation under the same parameters used during equilibration. To accelerate the simulation, we employed PMEMD.CUDA and saved the coordinates every 10 ps for subsequent analysis.

### 2.6 Essential dynamics

To understand the dynamics variation and atomic motion of the whole trajectories the similar conformations were clustered and presented as Principal components by using the principal component analysis approach (30). This approach clusters the simulation trajectories and has been widely used in large-scale data analysis. To further understand the stable and metastable states the two principal components i.e., PC1 and PC2 were used to determine the free energy landscape (FEL). It has been widely used to determine the lowest conformational state and variations as compared to the native conformation. For this purpose, CPPTRAJ was used and the *g_sham* module of Gromacs was used for the PC’s construction.

### 2.7 Calculation of binding free energies

The strength of a protein interacting with its biologically significant ligand/protein, or a small inhibitor significantly impacts the drug discovery and understanding of protein coupling mechanisms (31, 32). For protein-protein and protein-ligand complexes, this property is frequently represented by the binding free energies (BFE). In this work, it is calculated as the difference between the free energies of the bound aP2-CA33 complex (*G_complex, solvated_*) and the unbound states of aP2 (*G_aP2, solvated_*) and CA33 (*G_CA33, solvated_*), as shown in equation (i). For each complex, the hydrogen bonding and distances with energetic contribution were calculated from a relaxed structure. The following equation was used to calculate each term:


(i) $$\Delta G_{bind}=\;G_{\left(complex,\;\;solvated\right)}-\;G_{\left(aP2,\;\;solvated\right)}-\;G_{\left(CA33,\;\;solvated\right)}$$

This equation can be used to determine the contribution of interaction in the complex and can be expressed as equation (ii):


(ii) $$G=\;E_{Molecular\;Mechanics}-\;G_{solvated}-\;TS$$

This equation can be further restructured to calculate the specific energy term.


(iii) $$\Delta G_{bind}=\;\Delta E_{Molecular\;Mechanics}+\;\Delta G_{solvated}-\;\Delta TS=\;\Delta G_{vaccum}\;+\;\Delta G_{solvated}\;$$

(iv) $$\Delta E_{Molecular\;Mechanics}=\;\Delta E_{int}+\;\Delta E_{electrostatic}\;+\;\Delta E_{vdW}$$

(v) $$\Delta G_{solvated}=\;\Delta G_{Generalized\;born}+\Delta G_{surface\;area}$$

(vi) $$\Delta G_{surface\;area}=\;\gamma .SASA+b$$

(vii) $$\Delta G_{vaccum}=\;\Delta E_{Molecular\;Mechanics}-T\Delta S$$

Specifically, we represent the free energy associated with the total binding of proteins as ΔG_bind_ (iii, v, vii). This encompasses the cumulative gas phase energy, which consists of ΔE_internal_, ΔE_electrostatic_, and ΔE_vdW_, and is denoted as ΔE_MM_ (iv). The combined contributions from the polar (ΔGPB/GB) and nonpolar (ΔG_SA_) components of solvation are expressed as ΔG_sol_ (v). The conformational binding entropy, typically evaluated through normal-mode analysis, is denoted as -TΔS. The internal energy, resulting from various bonds, angles, and dihedrals in the molecular mechanics (MM) force field, is encapsulated in ΔE_internal_. Notably, in calculations involving MM/PBSA and MM/GBSA, this value remains consistently zero, as observed in the single trajectory of a complex calculation. ΔE_electrostatic_ and ΔE_vdW_ represent the electrostatic and van der Waals energies, respectively, computed using MM. Meanwhile, ΔGPB/GB signifies the polar contribution to the solvation-free energy, computed employing Poisson–Boltzmann (PB) or generalized Born (GB) methods. Lastly, ΔG_SA_ quantifies the nonpolar solvation-free energy, usually determined using a linear function based on solvent-accessible surface area (SASA) (vi). It’s worth noting that the calculation of conformational entropy is often omitted due to its computational expense and susceptibility to inaccuracies.

## 3 Results and discussion

### 3.1 CA33 mutants prediction and docking with aP2

**Figure 1:**
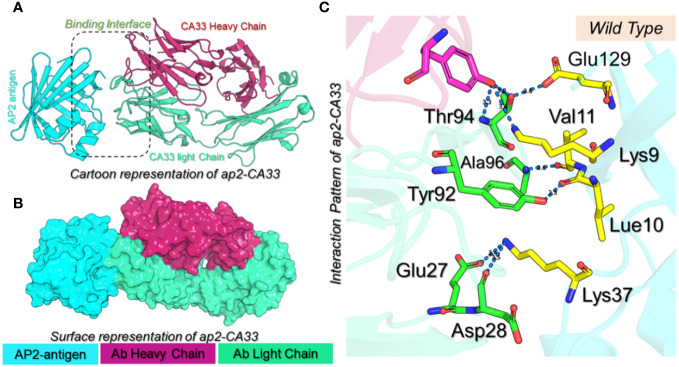
**(A)** Cartoon presentation of the aP2-CA33 complex. The aP2 antigen is shown in cyan color, the heavy chain of CA33 is shown in pomegranate color while the light chain is given in light green color. **(B)** shows the surface representation of the aP2-CA33 complex. **(C)** represents the interaction pattern for the aP2-CA33 complex, where the green color represents the L chain, magenta represents the H chain and the yellow represents aP2. The hydrogen bonding interactions are given in blue dashes with the bonding distances.

**Figure 2:**
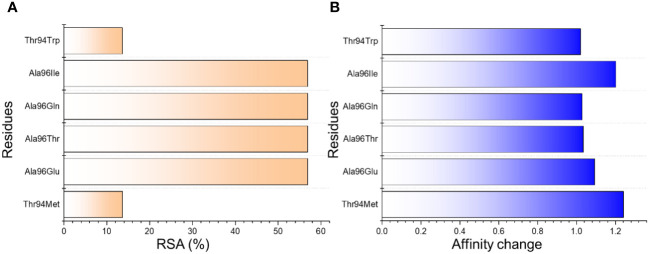
The predicted top mutants increase the binding affinity upon the substitution. **(A)** shows the relative surface area change in percent while **(B)** shows the affinity change due to each substitution.

Structural engineering of a protein has always been a great tool to increase the binding affinity and specificity for therapeutic purposes. Using graph-based signatures we generated the structural mutant of the L chain of the CA33 antibody. The complex as depicted in **Figures 1A, B** (cartoon and surface presentation) shows the binding of aP2 with the L and H chains of CA33. It was observed that the L chain only interacts directly with the binding residues of aP2 while the H chain comes in indirect contact with aP2 through non-bonded contacts. Before modeling the novel mutants, we analyzed the binding interface which revealed that the residues Lys9, Leu10, Val11, Thr56, Glu129, and Lys37 are involved in interaction with the aP2. Among these six hydrogen bonds were formed by Lys9-Thr94 (2.72Å), Lys9-Thr94 (3.26Å), Leu10-Tyr92 (2.26Å), Val11-Asp28 (2.89Å) and Glu129-Thr94 (2.55Å). The only salt bridge was reported between Lys37-Glu27 with a bonding distance of 3.41 Å. Considering this interaction paradigm we mutated the selected residues in the L chain of the antibody. We observed that mutating Tyr92 and Asp28 abolish the interactions while the others Glu27, Thr94, and Ala96 are non-essential contacts and favorable for substitutions that could result in higher binding affinity than the native complex. Among the predicted mutants 30 mutants were predicted to increase the binding affinity while the rest were predicted to decrease the binding affinity. We set a threshold of Predicted ΔΔG>1 that will be considered while the others should be considered as non-essential substitutions. Using this criterion, Thr94Met was observed to increase the binding affinity with the predicted ΔΔG of 1.24 to be the highest among all. The Ala96Gln replacement reported an affinity change in the predicted ΔΔG of 1.09 while the Ala96Thr, and Ala96Ile, Ala96Glu reported ΔΔG of 1.035, 1.202 and 1.02 respectively. The Thr94Trp substitution reported ΔΔG of 1.022 respectively. These top-scoring mutants were generated by using Chimera software and subjected to aP2-antibody docking using HADDOCK. The interaction pattern for the wild-type CA33 and aP2 is illustrated in **Figure 1C** while the predicted affinity change for top residues with RSA (accessible surface area) is provided in **Figures 2A, B**. The predicted Ramachandran plot (Clash Score, Ramachandran Favored/Outliers, rotamer Outliers) for dihedral angle analysis, and MolProbity Scores are summarized in **Supplementary Table S1**.

**Figure 3:**
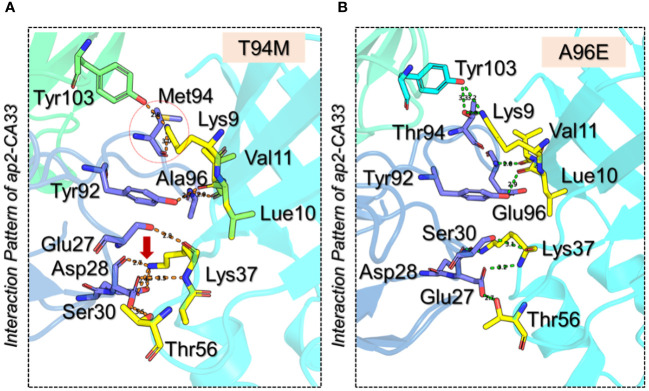
3D interaction paradigm for the T94M and A96E mutants in complex with aP2. **(A)** represent the interaction pattern of T94M with aP2. In this panel, the yellow sticks represent aP2, the blue sticks represent the L chain, and the green stick represents the H chain. **(B)** represents the interaction pattern of A96E with aP2. In this panel, the yellow sticks represent aP2, the blue sticks represent the L chain, and the cyan stick represents the H chain.

Next, we generated the mutants (**Figure 2**) that increase the binding affinity and modeled by using Modeler software embedded Chimera tool. To obtain the docking scores for the wild-type we submitted the native complex to the HADDOCK server and used a refinement option to get the results for the wild-type and use as a comparison for the further mutant’s selection. The HADDOCK server predicted the docking score for the wild-type of -364.90 ± 3.0 kcal/mol with the vdW (Van Der Waals) score of -184.70 ± 4.0 kcal/mol and the electrostatic energy of -498.00 ± 28.2 kcal/mol. The other parameters are provided in **Table 1**. Considering the total docking score of the wild-type (-364.90 ± 3.0 kcal/mol) the top-scoring mutants were selected based on this threshold. Among the selected mutants the two i.e., Ala96Leu reported a docking score of -363.50 ± 2.0 kcal/mol and Ala96Thr reported a docking score of -361.70 ± 5.3 kcal/mol which is a higher than the control (wild-type) and were excluded from the further analysis. The mutant Thr94Met predicted the best docking score among all. The docking score for the Thr94Met was calculated to be -372.00 ± 3.7 kcal/mol with ten hydrogen bonds and 2 salt bridges. A total of 51 non-bonded contacts were reported in this complex. In this complex Tyr103 established a hydrogen bond with Lys9 (2.8 Å) from the H chain while the L chain established the remaining nine hydrogen bonding contacts. Among these Glu27-Lys37 (2.8 Å), Asp28-Lys37 (2.7 Å), Ser30-Lys37 (3.5 Å), Ser30-Thr56 (3.5 Å), Tyr92-Leu10 (2.7 Å), Met94-Lys9 (2.99 Å), Ala96-Leu10 (2.9 Å) and Ala96-Val11 (2.9 Å) respectively. The only salt bridge was established between Lys37-Glu27 with a bonding distance of 2.70 Å. Interestingly the mutated residues Met94 directly interact with the aP2 and additional contacts have been established such as Ser30 interaction with Lys37 and Thr56. The interaction paradigm for the Thr94Met is shown in **Figure 3A**. For this complex the vdW was estimated to be -194.40 ± 6.1 kcal/mol while the electrostatic energy was calculated to be -459.7 ± 18.1 kcal/mol. In contrast to the native complex, this mutant presented a better vdW energy that particularly contributed to the robust binding of this mutant than the wild-type. On the other hand, the Ala96Glu with a docking score of -371.4 ± 1.9 was ranked as the second-best mutant that has a lower docking score than the wild-type. The rationale behind the increase in the docking is that this complex involved the highest number of non-bonded contacts with additional hydrogen bonds and the conserved salt bridge. The hydrogen bonding paradigm reported eight hydrogen bonds Lys9-Thr94 (2.83 Å), Leu10-Tyr92 (2.93 Å), Val11-Glu96 (2.79 Å), Lys37-Asp28 (3.17 Å), Lys37-Glu27 (2.85 Å), Lys37-Asp28 (2.84 Å) and Thr56-Asp28 (2.75 Å) respectively. The only salt bridge was established between Lys37-Glu27 with a bonding distance of 2.80 Å. Additionally, a hydrogen bond was also reported between the heavy chain Tyr103 and Lys9 residue of aP2. These additional hydrogen bonding contacts consequently increase the binding and neutralization of aP2 antigen through the recognition of essential immune epitopes. The vdW and electrostatic energies for this complex were calculated to be -192.30 ± 4.7 and -471.10 ± 17.8 kcal/mol respectively which are lower than the control native aP2-CA33 complex thus inducing stronger binding and neutralization. The interaction paradigm for the Ala96Glu is shown in **Figure 3B**. The docking scores and other parameters for these mutants are provided in **Table 1**.

**Table 1:** The predicted docking score for each substitution using HADDOCK. The bonding residues and distances for each complex.

Parameters	Wild-type-aP2	T94M-aP2	A96E-aP2	A96Q-aP2	T94W-aP2	A96L-aP2	A96T-aP2
**HADDOCK score**	-364.9 ± 3.0	-372.0 ± 3.7	-371.4 ± 1.9	-369.2 ± 2.3	-366.1 ± 2.0	-363.5 ± 2.0	-361.7 ± 5.3
**Cluster size**	20	20	20	20	20	20	20
**RMSd from the overall lowest-energy structure**	0.5 ± 0.3	0.5 ± 0.3	0.5 ± 0.3	0.6 ± 0.3	0.6 ± 0.3	0.5 ± 0.3	0.5 ± 0.3
**Van der Waals energy**	-184.7 ± 4.0	-194.4 ± 6.1	-192.3 ± 4.7	-190.9 ± 1.5	-192.9 ± 4.4	-189.3 ± 4.5	-185.7 ± 5.0
**Electrostatic energy**	-498.0 ± 28.2	-459.7 ± 18.1	-471.1 ± 17.8	472.8 ± 16.5	-437.0 ± 26.8	-432.3 ± 22.9	-495.6 ± 25.5
**Desolvation energy**	-80.7 ± 3.6	-85.6 ± 1.1	-84.9 ± 2.8	-83.8 ± 1.5	-85.8 ± 3.6	-87.8 ± 1.4	-76.9 ± 4.4
**Restraint’s violation energy**	0.2 ± 0.1	0.1 ± 0.1	0.2 ± 0.1	0.3 ± 0.3	0.2 ± 0.2	0.2 ± 0.1	0.3 ± 0.2
**Buried Surface Area**	4748.5 ± 44.2	4694.9 ± 49.8	4776.0 ± 48.2	4774.2 ± 67.2	4686.1 ± 32.8	4722.5 ± 62.5	4668.1 ± 76.5
**Z-Score**	0	0	0	0	0	0	0
**Dissociation constant (K_D_)**	1.2E^-8^	0.9E^-10^	1.1E^-9^	1.1E^-9^	1.2E^-6^	–	–
**Hydrogen Bonds**	Lys9-Thr94 (2.72Å), Lys9-Thr94 (3.26Å), Leu10-Tyr92 (2.26Å), Val11-Asp28 (2.89Å) and Glu129-Thr94 (2.55Å)	Glu27-Lys37 (2.8 Å), Asp28-Lys37 (2.7 Å), Ser30-Lys37 (3.5 Å), Ser30-Thr56 (3.5 Å), Tyr92-Leu10 (2.7 Å), Met94-Lys9 (2.99 Å), Ala96-Leu10 (2.9 Å) and Ala96-Val11 (2.9 Å)	Lys9-Thr94 (2.83 Å), Leu10-Tyr92 (2.93 Å), Val11-Glu96 (2.79 Å), Lys37-Asp28 (3.17 Å), Lys37-Glu27 (2.85 Å), Lys37-Asp28 (2.84 Å) and Thr56-Asp28 (2.75 Å)	Lys9-Thr94 (3.10 Å), Leu10-Tyr92 (3.23 Å), Val11-Gln96 (2.97 Å), Lys37-Asp28 (3.30 Å), Lys37-Asp28 (2.68 Å), Thr56-Asp28 (2.71 Å), Lys37-Asp28 (3.00 Å), Glu129-Thr94 (2.69 Å), and Glu129-Tyr103 (3.10 Å)	Glu27-Lys37 (2.7 Å), Asp28-Lys37 (2.7 Å), Asp28-Thr56 (3.3 Å), Ser30-Lys37 (3.1 Å), Tyr92-Leu10 (2.8 Å), Tyr103-Lys9 (2.8 Å) and Tyr103-Glu129 (3.4 Å)	–	–
**Salt bridges**	Lys37-Glu27 (3.41 Å)	Lys37-Glu27 (3.41 Å)	Lys37-Glu27 (2.80 Å)	Lys37-Glu27 (2.74 Å)	Lys37-Glu27 (2.74 Å)	–	–

**Figure 4:**
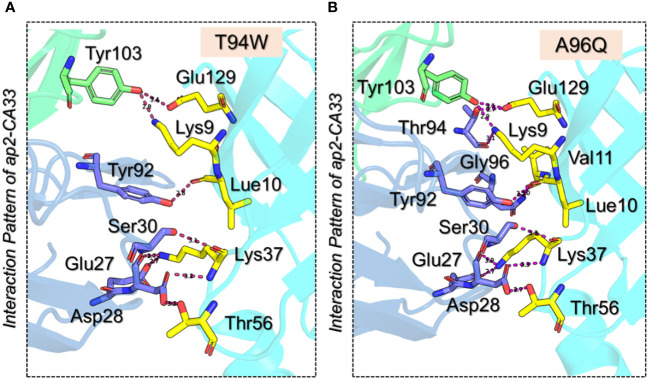
3D interaction paradigm for the T94W and A96Q mutants in complex with aP2. **(A)** represent the interaction pattern of T94W with aP2. In this panel, the yellow sticks represent aP2, the blue sticks represent the L chain, and the green sticks represent the H chain. **(B)** represents the interaction pattern of A96Q with aP2. In this panel, the yellow sticks represent aP2, the blue sticks represent the L chain, and the green sticks represent the H chain.

We further evaluated the binding patterns of T94W and A96Q mutants with aP2. The T94W with the docking score of -366.1 ± 2.0 kcal/mol reported eight hydrogen bonds involving Glu27-Lys37 (2.7 Å), Asp28-Lys37 (2.7 Å), Asp28-Thr56 (3.3 Å), Ser30-Lys37 (3.1 Å), Tyr92-Leu10 (2.8 Å), Tyr103-Lys9 (2.8 Å) and Tyr103-Glu129 (3.4 Å) respectively. Interestingly the heavy chain established two direct hydrogen bonds with the two residues of aP2 thus showing differential binding of this mutant. Moreover, the Ala96 interaction with Val11 was observed to be demolished while the extra contacts by the Ser30 can be seen in the complex. The Lys37-Glu27 (2.74 Å) salt bridge remained conserved here too. The vdW energy for this complex was observed to be -192.9 ± 4.4 kcal/mol while the electrostatic energy was -437.0 ± 26.8 kcal/mol respectively. The interaction pattern of T94W is shown in **Figure 4A**. On the other hand, the Ala96Gln reported a docking score of -369.2 ± 2.3 kcal/mol, vdW of -190.9 ± 1.5, and electrostatic energy of -472.8 ± 16.5 kcal/mol respectively. Investigation of the binding pattern revealed ten hydrogen bonds among which 2 were established by the H chain and the remaining 8 by the L chain. The other differences include the direct interaction of the H chain with the aP2. Among the hydrogen bonds Lys9-Thr94 (3.10 Å), Leu10-Tyr92 (3.23 Å), Val11-Gln96 (2.97 Å), Lys37-Asp28 (3.30 Å), Lys37-Asp28 (2.68 Å), Thr56-Asp28 (2.71 Å), Lys37-Asp28 (3.00 Å), Glu129-Thr94 (2.69 Å), and Glu129-Tyr103 (3.10 Å) respectively. The Lys37-Glu27 (2.74 Å) salt bridge remained conserved here too. The interaction pattern of A96Q is depicted in **Figure 4B**. The docking scores and other parameters for these mutants are summarized in **Table 1**. Overall, the current findings show that both the vdW and electrostatic energy terms are increased which consequently causes the robust binding of CA33 to the aP2. The current findings highlight the importance of protein engineering in the design of novel and effective therapeutics for the development of specific antibodies against T2DM.

### 3.2 Calculation of binding strength through K_D_

The binding strength was further validated by using the dissociation constant calculation based on the AI-powered algorithm trained with the experimental data. The results demonstrated that the K_D_ value for the wild-type was 1.2E^-8^ while for the T94M, the K_D_ was estimated to be 0.9E^-10^. For the T94W the K_D_ was estimated to be 1.1E^-9^, for the A96Q the K_D_ was computed to be 1.1E^-9^ and for the A96E the K_D_ was computed to be 1.2E^-6^. This shows the higher binding strength for the mutants except A96E and therefore demonstrates a robust immune response by interacting with aP2.

### 3.3 Dynamic stability assessment of the wild-type and mutant complexes

**Figure 5:**
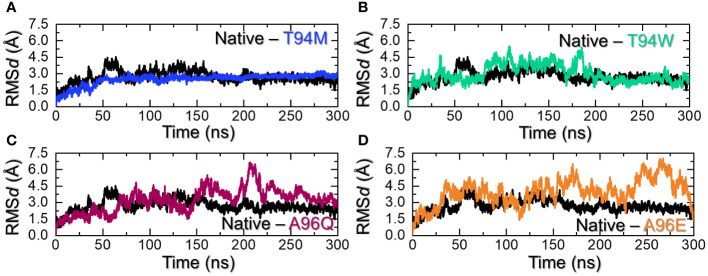
Dynamic stability assessment of the wild-type and mutants. **(A)** shows the RMS*d* graphs for the wild-type and T94M, **(B)** shows the RMS*d* graphs for the wild-type and T94W, **(C)** shows the RMS*d* graphs for the wild-type and A96Q while **(D)** shows the RMS*d* graphs for the wild-type and A96E.

Determining complex stability during simulation is an essential step towards the understanding of the pharmacological efficiency of a therapeutic molecule. It is considered as important for stable binding and therefore is necessary to estimate the system’s stability. Considering the importance of dynamic stability, we calculated root mean square deviation (RMS*d*) as a function of time using the simulation trajectory. As shown in **Figure 5A**, the wild-type antibody stabilized at 3.0 Å at 75ns. The complex initially demonstrated a higher RMS*d* with minor deviations, it stabilized and maintained the same level until the end of the simulation. An average RMS*d* for the wild-type was calculated to be 2.74 Å. On the other hand, the T94M stabilized at 2.25 Å at 37ns. The complex reported no significant perturbation and the average RMS*d* for this complex was calculated to be 2.40 Å. This indicates that the introduction of this mutant causes structural stabilization and thus the binding is further stabilized. Hence, this mutation is more favorable for enhancing the binding and instigation of a stronger immune response against aP2. Moreover, the T94W mutant reported a comparatively destabilized behavior than the T9M but was more stable than the wild-type at the end of the simulation. The trajectory started from 0 and reached 4.3 Å at 40ns. The complex then exhibited a stable behavior but after reaching 75ns the RMS*d* increased again and maintained the same level till 175ns. An abrupt rise in RMS*d* at 180ns was followed by a subsequent decline. After 190ns, the complex attained stability and maintained a uniform level until the end of the simulation. An average RMS*d* for this complex was calculated to be 2.95 Å. The RMS*d* results for the T94W are shown in **Figure 5B**. Interestingly, the A96Q and A96E substitutions were found to show dynamically unstable behavior with a reported RMS*d* higher than the wild-type and T94M/W mutants. For instance, the RMS*d* pattern for the A96Q reported significant structural perturbations with a higher RMS*d* level of 6.2 Å. The structure started with 1.5 Å until 50ns and then an abrupt increase/decrease was experienced. An average RMS*d* for the A96Q complex was estimated to be 3.24 Å. The A96E complex was observed to be the most destabilized complex with a reported RMS*d* of 6.5 Å. With significant structural perturbation, this complex maintained a higher RMS*d* level than all the complexes, with an average RMS*d* (4.58 Å) being observed. The RMS*d* graphs for the A96Q are shown in **Figure 5C** while the RMS*d* graph for the A96E is depicted in **Figure 5D**. It can be observed that the T94M is the most stable substitution which increases the binding stability throughout the simulation while the T94W also exhibited comparatively a dynamically stable behavior. The superimposed structures of each complex retrieved at different time intervals were further compared with the native to understand the structural variations. As shown in **Supplementary Figure S1**, it can be noted that the interface in all the complexes remains intact while the tail of the L chain folds and unfolds inward and outward to cause deviation from the native structure. Moreover, the flipping of the beta sheets in the aP2 also causes deviation from the native structure. This shows that the aP2-CA33 remains bound during simulation however the movement of some secondary structural elements causes the drift in the RMS*d* pattern. In sum, these two substitutions are more favorable for the enhanced and stabilized binding of the CA33 antibody than the A96E and A96Q and therefore should be further investigated for clinical purposes.

### 3.4 Structural compactness assessment

**Figure 6:**
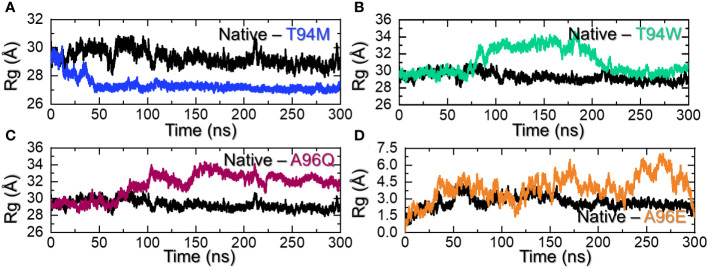
Structural compactness assessment of the wild-type and mutants. **(A)** shows the R*g* graphs for the wild-type and T94M, **(B)** shows the R*g* graphs for the wild-type and T94W, **(C)** shows the R*g* graphs for the wild-type and A96Q while **(D)** shows the R*g* graphs for the wild-type and A96E.

Calculation of the structural compactness by using a radius of gyration (R*g*) over the simulation time is an important parameter that determines the binding and unbinding events during the simulation. It is an essential step to determine the pharmacological potential of a therapeutic molecule. Considering the application of R*g* in determining structural stability and compactness, we also calculated Rg using the simulation trajectory. Interestingly, the R*g* results for the wild-type aligned with the RMS*d* results. The R*g* started from 29.80 Å and steadily decreased over time. The highest Rg value was observed at 70ns and then a continuous decline in the R*g* value was observed. An average Rg for the wild-type was calculated to be 29.85 Å. On the other hand, the R*g* for T94M mutant started at 30.0 Å and continued to decrease till 26.8Å at 50ns. The complex then reported a uniform straight graph for Rg values and no deviation was observed. This indicates that the complex maintained a rigid and stabilized compact structure and therefore had minimal unbinding events during the simulation. The R*g* results strongly align with the RMS*d* results, with stability maintained throughout the simulation. An average R*g* for the T94M complex was estimated to be 27.0 Å as shown in **Figure 6A**. The T94W initially reported a lower Rg (30.0 Å) behavior by keeping the R*g* at 30.0 Å up to 75ns. The R*g* then gradually increased and continued to report a similar behavior until 225ns. Like the RMS*d* results, the Rg also maintained a stable and lower level during the last part of the simulation. The increase in the Rg pattern determines the unwinding of the tail of the CA3 which causes a significant increase in the protein size. The R*g* for the T94W is shown in **Figure 6B**. Interestingly, the A96Q comparatively reported a stabilized protein size during the first 75ns and then gradually increased up to 32.0 Å. This R*g* level was maintained for the remaining simulation time showing the unwinding of the CA33 tail and then rewind. An average R*g* for the A96Q was calculated to be 31.5 Å (**Figure 6C**). The Rg results for the A96E also reported a similar behavior to the findings of RMS*d*. The R*g* remained higher than all the complexes. This complex maintained an Rg level of ~34.50 Å throughout the simulation. An average R*g* for the A96E was calculated to be 34.45Å (**Figure 6D**). Overall, these findings strongly corroborate with the RMS*d* and show that T94M and T94W are the most favorable that not only increase the binding but also increase the stability. Interestingly, the higher binding mutant remained the most compact avoiding the unbinding events while the three other substitutions i.e., T94W, A96Q, and A96E caused structural instability. Thus, substitutions that increase the structural stability increase the binding significantly.

### 3.5 Hydrogen bonding analysis

**Figure 7:**
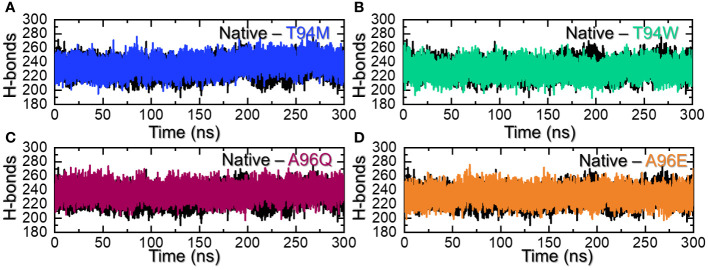
Hydrogen bonding analysis of the wild-type and mutants. **(A)** shows the H-bonds graphs for the wild-type and T94M, **(B)** shows the H-bonds graphs for the wild-type and T94W, **(C)** shows the H-bonds graphs for the wild-type and A96Q while **(D)** shows the H-bonds graphs for the wild-type and A96E.

Hydrogen bonding calculation is one of the key assessments that help in determining the pharmacological potential of a drug or inhibitor. It is an essential approach to reveal the potency and binding strength of the interacting molecules. This approach has been widely applied to understand the pharmacological mechanism of a particular drug, and the interaction mechanism of two or more proteins to reveal the mechanism of a disease or bio-catalytic process (33–37). Considering the essential role of this approach, we used a similar approach to calculate the total number of hydrogen bonds in each complex. The average number of hydrogen bonds in each complex was calculated to be 231 in the wild-type, 236 in the T94M, 229 in the T94W, 232 in the A96Q, and 231 in A96E. It can be observed that the hydrogen bonds in the predicted mutants are more than the wild-type thus implying that these mutants increase the binding. Although the number of bonds is increased in the three mutants T94M is the more favorable substitution that increases the binding stability with the number of hydrogen bonds. The hydrogen bonding results for all the complexes are presented in **Figures 7A–D**. Additional information about the hydrogen bonding, distances, and half-life information are summarized in **Supplementary Table S2**.

### 3.6 Root mean square fluctuation calculation

**Figure 8:**
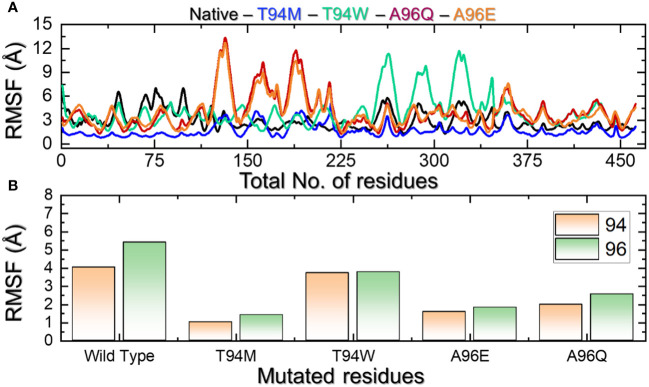
**(A)** Residue’s flexibility analysis of the wild-type and mutants. All the complexes are differently colored. **(B)** shows the RMSF pattern for the mutated residues in each complex.

Residue fluctuation indexing is an essential factor in determining the role of particular residues in molecular recognition, protein inhibition, ligand recognition, and opening and closing switches. For instance, this approach has been widely used to determine the impact of different mutations on the binding and internal fluctuation of different receptors (38). Herein, we also calculated residual flexibility using the simulation trajectory. The RMSF results presented in **Figure 8A** demonstrate that the internal fluctuation of the aP2 has been stabilized and thus minimal fluctuations are produced by the wild-type and T94M complexes while the other complexes have produced higher fluctuations. The regions 35-225 and 230-335 determined major fluctuations in the T94W, A96Q, and A96E. We further dissected the RMSF profiles of each mutated residue in each complex. The results shown in **Figure 8B** indicate that the mutated residues demonstrated higher fluctuation than the wild-type and therefore result in better conformational optimization for enhanced binding. Interestingly, the RMSF results also corroborate with the binding results and indicate that wild-type and T94M are better immune response-provoking agents than the other mutants.

### 3.7 Principal component analysis for trajectories motions clustering

**Figure 9:**
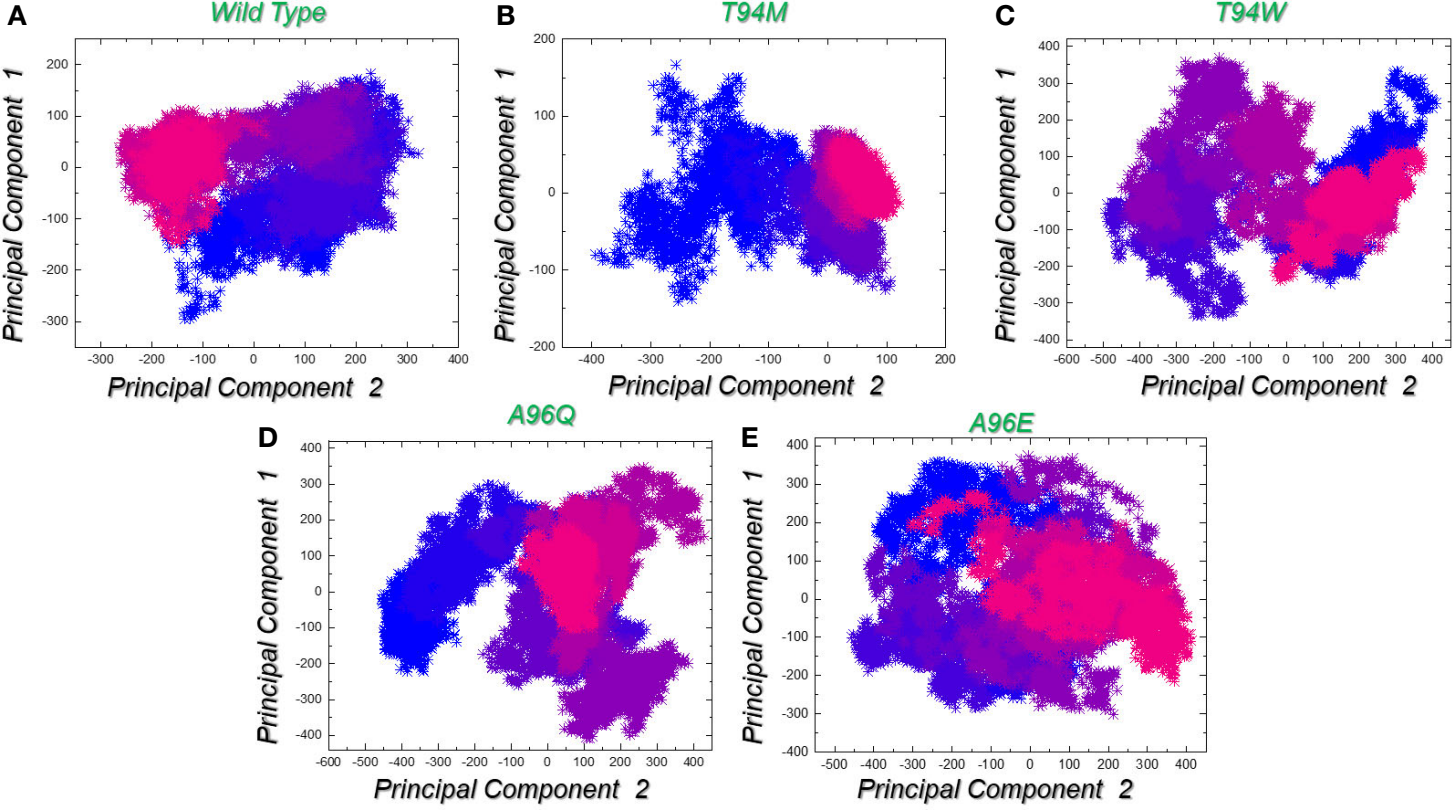
Trajectories clustering and motion using principal component analysis (PCA). **(A)** represents the trajectory distribution for the wild-type complex in X and Y dimensions given as PC1 and PC2. **(B)** represents the trajectory distribution for the T94M complex in X and Y dimensions given as PC1 and PC2. **(C)** represents the trajectory distribution for the T94W complex in X and Y dimensions given as PC1 and PC2. **(D)** represents the trajectory distribution for the A96Q complex in X and Y dimensions given as PC1 and PC2. **(E)** represents the trajectory distribution for the A96E complex in X and Y dimensions given as PC1 and PC2.

The analysis of data distribution within the component space yields valuable insights into the fundamental dynamics of the underlying system. Notably, both the wild-type and T94M had comparable patterns of constraint and restricted motion across each principal component. It further shows that these two systems are more stable and controlled in these dimensions. The conformational space is divided into two states i.e., the pink color which is separated by the purple color (transition state) from the blue color. On the other hand, the T94W, A96Q, and A96E determined differential trajectories clustering and therefore presented an unstable state for each complex. This indicates that mutant T94M behaves more like the wild-type but presents favorable variations that cause more robust binding of T94M than the control. These findings also corroborate the residues’ flexibility and docking results. The PCA graphs are presented in **Figures 9A–E**.

### 3.8 Free energy landscape analysis

**Figure 10:**
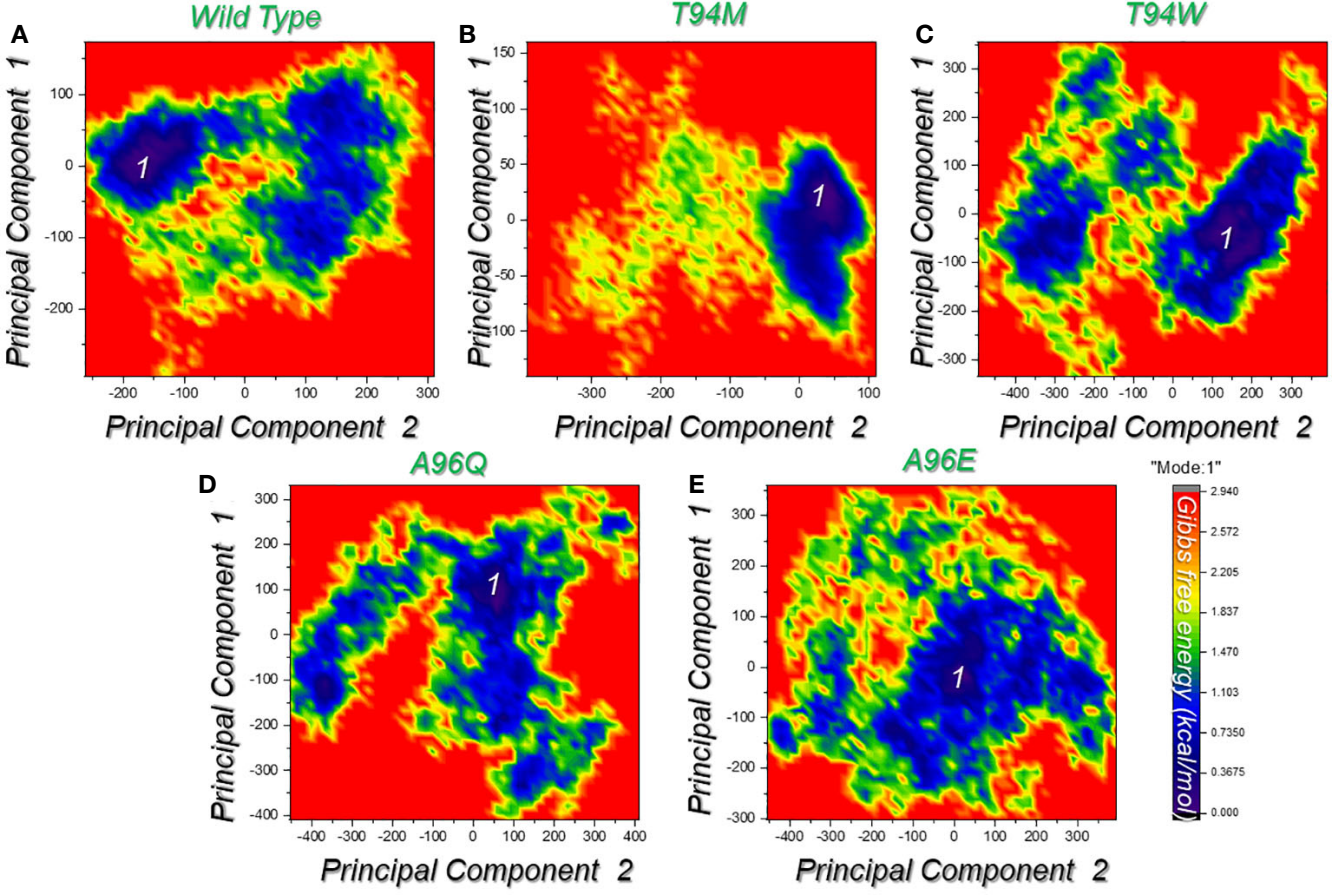
Free energy landscape (FEL) analysis of the wild-type and the designed mutated antibodies. **(A)** represents the FEL for the wild-type complex in X and Y dimensions given as PC1 and PC2. **(B)** represents the FEL for the T94M complex in X and Y dimensions given as PC1 and PC2. **(C)** represents the FEL for the T94W complex in X and Y dimensions given as PC1 and PC2. **(D)** represents the FEL for the A96Q complex in X and Y dimensions given as PC1 and PC2. **(E)** represents the FEL for the A96E complex in X and Y dimensions given as PC1 and PC2. Each graph represents the only conformational state attained by each complex.

In the context of molecular mechanics and simulation, the free energy landscape is used to understand and visualize the energy landscape of each system. It provides a visual presentation of the relationship between the potential energy and its collective variables. It determines the possible lowest energy configuration state and determines the protein folding. All the complexes presented a single metastable (lowest energy state) during the simulation which indicates that the system does not readily transit through multiple conformations. This demonstrates limited structural variability and underscores the therapeutic antibody’s efficacy against aP2. The FEL graphs are presented in **Figures 10A–E**.

### 3.9 Binding free energy analysis

**Figure 11:**
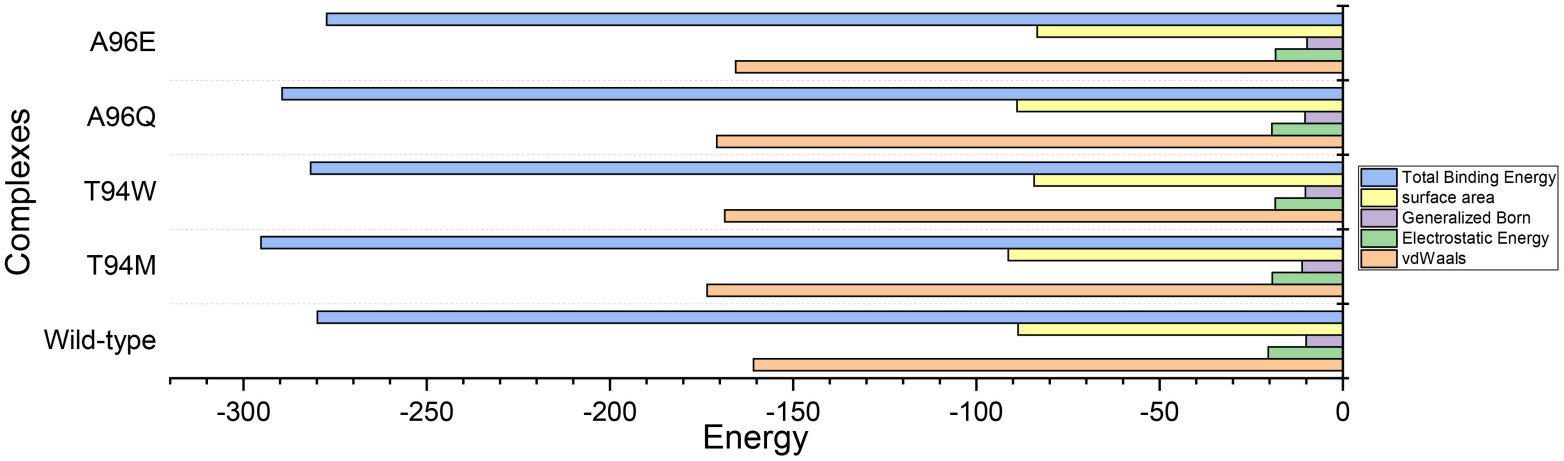
Total binding free energy results for each complex using the MM-GBSA approach. All the energies are given in kcal/mol.

We calculated the binding free energy for each complex which revealed that vdW values of -160.82 kcal/mol, -173.49 kcal/mol, -165.69 kcal/mol, -170.83 kcal/mol, -168.67 kcal/mol were calculated for wild-type, and T94M, A96Q, and A96E mutants, respectively. This indicates that the rise in the number of hydrogen bonds leads to a corresponding increase in the vdW energy within each complex, causing the binding affinity to strengthen. On the other hand, the electrostatic energy calculations showed Elec values of -20.36 kcal/mol, -19.27 kcal/mol, -18.39 kcal/mol, -19.35 kcal/mol, -18.48 kcal/mol for wild-type, T94M, T94W, A96Q, and A96E mutant, respectively. To provide conclusive evidence on the role of the introduced mutations and their impact on the binding, we calculated the total binding free energy for each complex to accurately evaluate the binding strength of each complex. The results strongly corroborate with the docking scores and dissociation constant (K_D_) results. The TBE for the wild-type was computed to be -279.84 kcal/mol, for the T94M the highest binding free energy was estimated to be -295.22 kcal/mol. For the T94W, the binding free energy was computed to be -281.67 kcal/mol, and for the A96Q the TBE was -289.44 kcal/mol while for the A96E the TBE was estimated to be -277.29 kcal/mol. This shows that the predicted substitutions strongly corroborate with the hypothesis of affinity-increasing mutants that consequently cause an enhanced binding of the CA33-engineered antibody to the aP2 antigen. The binding free energy results for each complex are shown in **Figure 11**. The specific energy contribution is summarized in **Supplementary Table S2**.

## 4 Conclusions

The current study utilized structure-guided engineering strategies to enhance the CA33 antibody, leveraging graph-signature-based algorithms for rationale antibody design. The mutational landscape was subjected to a thorough examination, which revealed the presence of only four substitutions that were found to be significant. These alterations include T94M, T94W, A96Q, and A96E. Additional validation was conducted using post-prediction molecular simulations, which confirmed that the T94M substitution was the most favorable. Significantly, this change not only enhanced the docking score but also demonstrated exceptional stability throughout the simulation. To bolster the robustness of our results, we employed K_D_ estimates to quantify the binding affinity, introducing an additional level of validation to our investigation. Future directions for this research involve investigating similar antibodies and exploring diverse diabetes-related biotargets. Analyzing additional antibodies using similar structurally guided engineering approaches promises a more thorough understanding of potential improvements. Expanding the study with a broader range of mutations and rigorous experimental validation can address the limitations and enhance the robustness of the findings. A comprehensive exploration of various diabetes-related biotargets will contribute to a holistic approach to antibody design. Although the findings of this study have the potential to offer significant insights into the strategic design of diabetes-targeting antibodies, collaborative efforts with experimentalists for *in vitro* and *in vivo* validations are anticipated, paving the way for the translation of these insights into clinical trials and practical applications.

## Data availability statement

The original contributions presented in the study are included in the article/**Supplementary Material**. Further inquiries can be directed to the corresponding author.

## Author contributions

AK: Conceptualization, Data curation, Formal Analysis, Investigation, Methodology, Software, Validation, Visualization, Writing – original draft, Writing – review & editing. MZ: Conceptualization, Data curation, Investigation, Methodology, Visualization, Writing – original draft. AM: Conceptualization, Data curation, Formal Analysis, Funding acquisition, Investigation, Methodology, Project administration, Resources, Software, Supervision, Validation, Visualization, Writing – original draft, Writing – review & editing. AA: Conceptualization, Data curation, Formal Analysis, Funding acquisition, Investigation, Methodology, Project administration, Resources, Software, Supervision, Validation, Visualization, Writing – original draft, Writing – review & editing.
