# Supplementary material for: Structure-guided engineering and molecular simulations to design a potent monoclonal antibody to target aP2 antigen for adaptive immune response instigation against type 2 diabetes
Source: Front Immunol. 2024 Mar 8;15:1357342. doi: 10.3389/fimmu.2024.1357342 (PMC10960362; doi:10.3389/fimmu.2024.1357342)
Supplement: Supplementary file 1 [file DataSheet_1.docx]

**Supplementary Materials**

**Structure-guided engineering and molecular simulations to design a potent monoclonal antibody to target aP2 antigen for adaptive immune response instigation against type 2 diabetes**

Abbas Khan^1^, Muhammad Ammar Zahid^1^, Anwar Mohammad^2^, Abdelali Agouni^1*^

^1^Department of Pharmaceutical Sciences, College of Pharmacy, QU Health, Qatar University, P.O. Box 2713, Doha, Qatar

^2^Department of Biochemistry and Molecular Biology, Dasman Diabetes Institute, Dasman, Kuwait

***Corresponding author**

Prof. Abdelali Agouni, Department of Pharmaceutical Sciences, College of Pharmacy, Qatar University, P.O. Box 2713, Doha, Qatar. **Tel:** +974 4403 5610; **Email:** [aagouni@qu.edu.qa](mailto:aagouni@qu.edu.qa)

**Table S1.** MolProbity and Ramachandran plot analysis for the wild-type and mutant systems.

| Wild-Type | | | | |
| --- | --- | --- | --- | --- |
| Protein | Poor rotamers | 31 | 16.40% | Goal: <0.3% |
| Geometry | Favored rotamers | 138 | 73.02% | Goal: >98% |
|  | Ramachandran outliers | 2 | 0.93% | Goal: <0.05% |
|  | Ramachandran favored | 195 | 91.12% | Goal: >98% |
|  | Rama distribution Z-score | -3.11±0.48 | | Goal: abs(Z score) < 2 |
|  | Cβ deviations >0.25Å | 0 | 0.00% | Goal: 0 |
|  | Bad bonds: | 0 / 1700 | 0.00% | Goal: 0% |
|  | Bad angles: | 0 / 2313 | 0.00% | Goal: <0.1% |
| Peptide Omegas | Cis Prolines: | 02-Nov | 18.18% | Expected: ≤1 per chain, or ≤5% |
| Low-resolution Criteria | CaBLAM outliers | 7 | 3.30% | Goal: <1.0% |
|  | CA Geometry outliers | 0 | 0.00% | Goal: <0.5% |
| Additional validations | Chiral volume outliers | 0/258 | |  |
| T94M | | | | |
|  |  |  |  |  |
| Protein | Poor rotamers | 33 | 17.46% | Goal: <0.3% |
| Geometry | Favored rotamers | 131 | 69.31% | Goal: >98% |
|  | Ramachandran outliers | 2 | 0.93% | Goal: <0.05% |
|  | Ramachandran favored | 198 | 92.52% | Goal: >98% |
|  | Rama distribution Z-score | -2.74±0.51 | | Goal: abs(Z score) < 2 |
|  | Cβ deviations >0.25Å | 0 | 0.00% | Goal: 0 |
|  | Bad bonds: | 0 / 1701 | 0.00% | Goal: 0% |
|  | Bad angles: | 0 / 2313 | 0.00% | Goal: <0.1% |
| Peptide Omegas | Cis Prolines: | 02-Nov | 18.18% | Expected: ≤1 per chain, or ≤5% |
| Low-resolution Criteria | CaBLAM outliers | 6 | 2.80% | Goal: <1.0% |
|  | CA Geometry outliers | 0 | 0.00% | Goal: <0.5% |
| Additional validations | Chiral volume outliers | 0/257 | |  |
| T94W | | | | |
|  |  |  |  |  |
| Geometry | Favored rotamers | 137 | 72.49% | Goal: >98% |
|  | Ramachandran outliers | 2 | 0.93% | Goal: <0.05% |
|  | Ramachandran favored | 201 | 93.93% | Goal: >98% |
|  | Rama distribution Z-score | -3.80±0.47 | | Goal: abs(Z score) < 2 |
|  | Cβ deviations >0.25Å | 0 | 0.00% | Goal: 0 |
|  | Bad bonds: | 0 / 1701 | 0.00% | Goal: 0% |
|  | Bad angles: | 0 / 2315 | 0.00% | Goal: <0.1% |
| Peptide Omegas | Cis Prolines: | 02-Dec | 16.67% | Expected: ≤1 per chain, or ≤5% |
| Low-resolution Criteria | CaBLAM outliers | 2 | 0.90% | Goal: <1.0% |
|  | CA Geometry outliers | 1 | 0.47% | Goal: <0.5% |
| Additional validations | Chiral volume outliers | 0/257 | |  |
| A96E | | | | |
| Protein Geometry | Favored rotamers | 84 | 74.34% | Goal: >98% |
|  | Ramachandran outliers | 0 | 0.00% | Goal: <0.05% |
|  | Ramachandran favored | 124 | 96.12% | Goal: >98% |
|  | Rama distribution Z-score | -2.68±0.68 | | Goal: abs(Z score) < 2 |
|  | Cβ deviations >0.25Å | 0 | 0.00% | Goal: 0 |
|  | Bad bonds: | 0 / 1028 | 0.00% | Goal: 0% |
|  | Bad angles: | 0 / 1380 | 0.00% | Goal: <0.1% |
| Peptide Omegas | Cis Prolines: | 0 / 1 | 0.00% | Expected: ≤1 per chain, or ≤5% |
| Low-resolution Criteria | CaBLAM outliers | 1 | 0.80% | Goal: <1.0% |
|  | CA Geometry outliers | 0 | 0.00% | Goal: <0.5% |
| Additional validations | Chiral volume outliers | 0/161 | |  |
| A96Q | | | | |
| Protein | Poor rotamers | 9 | 7.96% | Goal: <0.3% |
| Geometry | Favored rotamers | 85 | 75.22% | Goal: >98% |
|  | Ramachandran outliers | 0 | 0.00% | Goal: <0.05% |
|  | Ramachandran favored | 123 | 95.35% | Goal: >98% |
|  | Rama distribution Z-score | -3.43±0.63 | | Goal: abs(Z score) < 2 |
|  | Cβ deviations >0.25Å | 0 | 0.00% | Goal: 0 |
|  | Bad bonds: | 0 / 1028 | 0.00% | Goal: 0% |
|  | Bad angles: | 0 / 1380 | 0.00% | Goal: <0.1% |
| Peptide Omegas | Cis Prolines: | 0 / 1 | 0.00% | Expected: ≤1 per chain, or ≤5% |
| Low-resolution Criteria | CaBLAM outliers | 5 | 3.90% | Goal: <1.0% |
|  | CA Geometry outliers | 0 | 0.00% | Goal: <0.5% |
| Additional validations | Chiral volume outliers | 0/161 | |  |

**Table S2.** Residue-wise energetic contribution, bonding distance, and estimated half-life for the minimized and relaxed structure.

| Residues | Energy | Distance (Å) | Half-life |
| --- | --- | --- | --- |
| Wild Type | | | |
| Lys9-Thr94 | -9.70 | 2.99 | 76% |
| Glu27-Lys37 | -8.73 | 3.10 | 64% |
| Ser30-Lys37 | -2.50 | 2.76 | 49% |
| Lys37-Glu27 | -18.13 | 2.96 | 61% |
| Lys37-Asp28 | -12.3 | 3.07 | 36% |
| Tyr92-Leu10 | -3.4 | 2.73 | 53% |
| Ala96-Val11 | -2.7 | 2.92 | 28% |
| T94M | | | |
| Lys9-Tyr92 | -14.10 | 2.76 | 64% |
| Lys9-Met94 | -6.80 | 3.01 | 79% |
| Glu27-Lys37 | -12.63 | 2.75 | 68% |
| Ser30-Lys37 | -2.60 | 2.75 | 42% |
| Tyr32-Ala131 | -2.00 | 2.78 | 14% |
| Lys37-Glu27 | -19.23 | 2.75 | 68% |
| Lys37-Asp28 | -8.40 | 2.86 | 6% |
| Lys50-Ala131 | -15.96 | 2.70 | 2% |
| Thr56-Asp28 | -3.10 | 2.66 | 11% |
| Tyr92-Leu10 | -2.50 | 2.57 | 37% |
| Ala96-Val11 | -4.50 | 2.81 | 34% |
| Tyr103-Glu129 | -4.60 | 2.60 | 8% |
| Lys112-Tyr51 | -1.00 | 2.95 | 14% |
| Ala131-Lys50 | -6.76 | 2.70 | 8% |
| T94W | | | |
| Lys9-Met94 | -9.70 | 2.99 | 76% |
| Glu27-Lys37 | -8.73 | 3.10 | 58% |
| Ser30-Lys37 | -2.50 | 2.76 | 31% |
| Lys37-Glu27 | -18.13 | 2.96 | 47% |
| Lys37-Asp28 | -12.3 | 3.07 | 10% |
| Gln38-Gln38 | -3.90 | 2.97 | 4% |
| Tyr92-Leu10 | -3.40 | 2.73 | 23% |
| Ala96-Val11 | -2.70 | 2.92 | 29% |
| A96E | | | |
| Lys9-Thr94 | -9.20 | 2.97 | 65% |
| Ser12-Glu96 | -0.70 | 3.33 | 38% |
| Ser13-Glu96 | -9.21 | 2.72 | 72% |
| Glu27-Lys37 | -10.35 | 2.92 | 43% |
| Tyr32-Ala131 | -1.91 | 2.70 | 8% |
| Lys37-Glu27 | -16.45 | 2.85 | 40% |
| Lys37-Asp28 | -14.70 | 2.82 | 6% |
| Gln38-Gln38 | -2.40 | 3.05 | 5% |
| Lys50-Ala131 | -24.51 | 2.76 | 3% |
| Thr56-Asp28 | -3.00 | 2.59 | 14% |
| Tyr92-Leu10 | -3.3 | 2.64 | 28% |
| Thr94-Glu129 | -2.1 | 2.52 | 26% |
| Glu96-Val11 | -0.8 | 2.87 | 7% |
| Asn97-Ala131 | -2.40 | 2.91 | 2% |
| Tyr103-Arg130 | -3.20 | 2.77 | 4% |
| Ala131-Lys50 | -6.219 | 2.76 | 3% |
| A96Q | | | |
| Lys9-Thr94 | -5.10 | 2.93 | 79% |
| Glu27-Lys37 | -12.4 | 2.77 | 58% |
| Tyr32-Asn39 | -2.80 | 2.80 | 24% |
| Lys37-Glu27 | -25.20 | 2.76 | 39% |
| Lys37-Asp28 | -13.60 | 2.97 | 11% |
| Lys50-Ala131 | -24.51 | 2.63 | 4% |
| Ser55-Ser30 | -1.00 | 2.94 | 7% |
| Thr56-Asp28 | -2.30 | 2.56 | 9% |
| Tyr92-Leu10 | -3.30 | 2.65 | 37% |
| Thr94-Glu129 | -2.20 | 2.54 | 20% |
| Gln96-Val11 | -7.80 | 2.97 | 47% |
| Ala131-Lys50 | -7.41 | 2.63 | 6% |


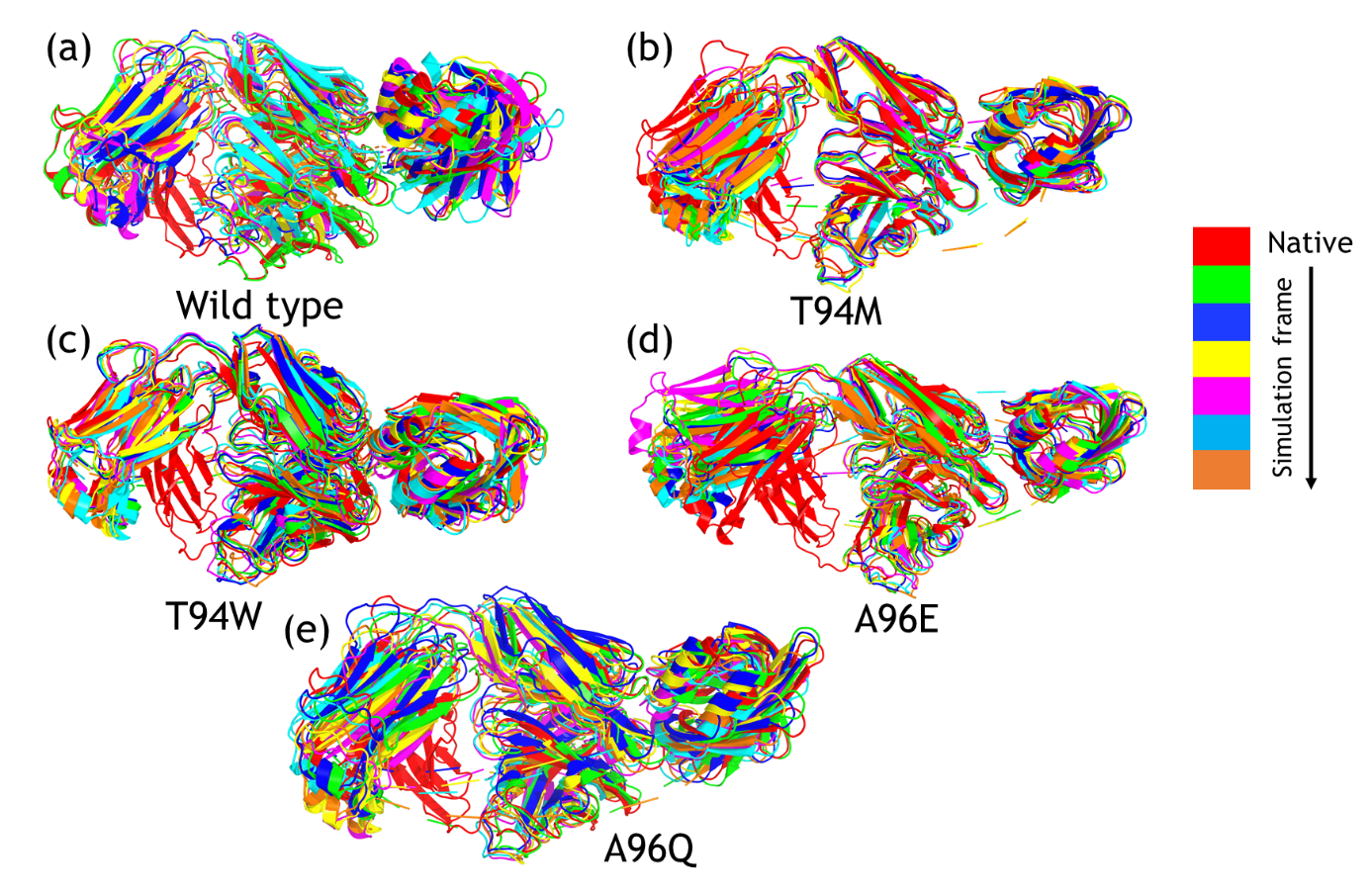


**Figure S1.** superimposed structures of the wild-type and mutant complexes at different time intervals retrieved from the simulation trajectory.
